# Supplementary material for: Evolutionary Analysis of TCGA Data Using Over- and Under- Mutated Genes Identify Key Molecular Pathways and Cellular Functions in Lung Cancer Subtypes
Source: Cancers (Basel). 2022 Dec 20;15(1):18. doi: 10.3390/cancers15010018 (PMC9817988; doi:10.3390/cancers15010018)
Supplement: Supplementary file 1 [file cancers-15-00018-s001.zip › cancers-2014646-supplementary.pdf]

Supplementary Materials

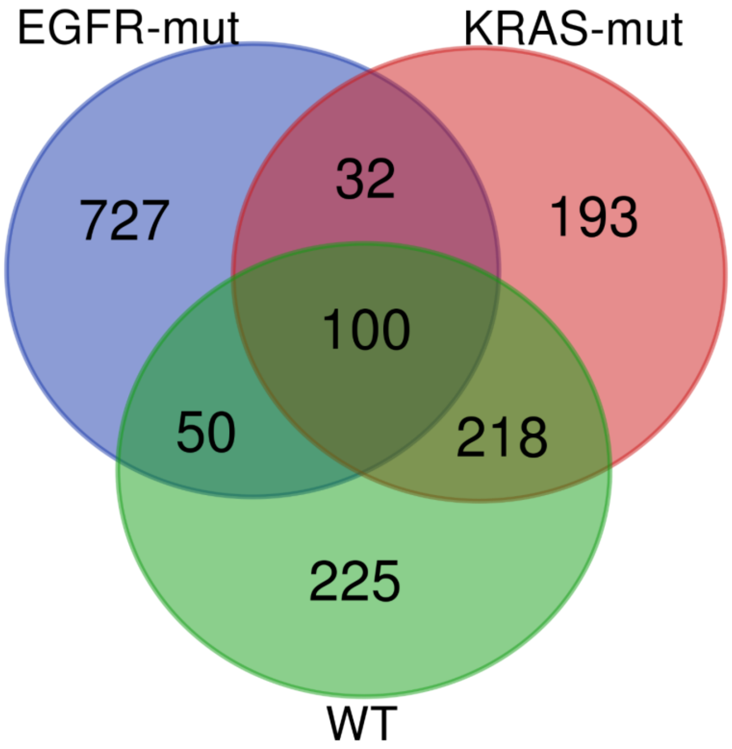

Figure S1. Evolutionarily selected genes defined as an observed frequency >2 standard deviations above the neutral line. As noted in the text, this approach significantly overestimates the evolutionary value of mutation and underestimates conservation in the *EGFR*-mut cancers because of the small cohort size and low mutation rate. For the analysis of gene mutations and conservations in *EGFR*-mut cancers, we use the criteria outlined below.

Names	Total	Elements
EGFR-mut KRAS-mut WT	100	*CTNND2 LILRB2 CACNA2D1 FAM5C HEPH BAI3 NLRP3 MDGA2 OR14A16 LRFN5 PRDM9 CACNA1E CDH10 TMEM200A OR2W3 HDAC9 OR10J3 FSTL5 ZFPM2 ZFHX4 CSMD3 PCDHB2 OR2AK2 LRRIQ3 USH2A LPPR4 PAPPA2 PCDH15 ABCB5 ZNF479 DCAF8L2 KLHL4 CNTN5 ZNF536 CSMD1 FERD3L OR2M4 TRIM58 TMPRSS15 OR5T2 DCAF12L2 CNBD1 CACNA1C ZNF676 OR6F1 KCNK2 SORCS1 PCDHGA2 EPHA3 PCDHB7 NLGN4X C1orf173 LRRTM4 KLHL1 FAM47C OR5D16 NELL1 C15orf2 SORCS3 TSHZ3 GRM5 EPHA6 NRG3 OR4K17 DCAF4L2 SPTA1 FAM47A ZP4 ST6GAL2 RYR2 OR1C1 FAM71B KCNA5 NTNG1 ZNF804B GABRA4 SLITRK2 TLR4 TPTE CNTNAP5 TP53 CD1E STK11 FLG RIMS2 OVCH1 ADCY2 OR4Q3 CDH12 TECRL PSG11 CDKN2A C18orf34 XIRP2 TNR CRB1 FMN2 OR5D13 XYLT1 NLRP14*
EGFR-mut KRAS-mut	32	*ERBB4 HOXA13 TBX5 RBM10 VN1R2 KRTAP11-1 INSC NLRP5 KIF4B BTK COL6A2 GRM6 TAS2R1 PCDHGB2 CPA4 TRPC4 GP2 FAM123B FOXG1 KCNH1 PCDHA5 PTPRN2 OR2A12 FAM83B ITIH6 OTUD6A DSG3 OTOGL ARHGAP6 OR2A14 PCDHGB3 OR1S1*
EGFR-mut WT	50	*SALL1 PDE1C HOXA1 OR10AG1 PSG8 POU3F4 FOLH1 OR2T1 PCDHB8 HOXA3 OR2W5 GPR158 SELP OR8K3 IRX1 PYHIN1 OR2T2 CDH6 CYYR1 PROS1 ADCY8 GRIN2A MYO7B CCDC129 ITGA4 SEMA5B NFASC MMD2 KCNH8 CLCN1 C16orf82 OR2B11 MUC17 OR4S2 IRX2 DUSP27 PDHA2 KCNH5 TYR PCDHA1 GABRA5 PDGFRA ATRNL1 SEMA5A ADAMTS5 MARCH1 PCDHGA3 MS4A14 HECW1 SPEF2*
KRAS-mut WT	218	*OR2T33 MPPED2 OR51V1 COL22A1 ZNF385D OR5L1 ZEB1 OR5AS1 HCN1 C7orf58 DOCK2 ZNF208 MAGEC3 OR5D14 KCNJ18 OR4C46 GRM8 ZCCHC5 SLC8A1 OR4A15 VGLL3 GNAS OR8J3 FBN2 GALNT13 ZNF257 SNTG1 PABPC5 BCHE RBFOX1 HCRTR2 MMP16 LRRC4C OR4M2 OR4N5 PDZRN3 NDST4 ADAMTS16 POTEG POM121L12 FAM135B GRIK2 ZNF716 OR2G2 SLITRK4 FAM47B EPHA5 C1orf129 ACTRT1 SPAM1 PCDHB11 ITGA8 ADAMTS2 MYH1 GABRG1 TIAM1 ZIC4 PCDHA2*

		KCNA4 OR2T12 SLC39A12 OR4N4 GPR112 OR8I2 KCNB2 SATB2 FAM75D1 CDH18 CHL1 ZIC1 MAGEC1 ANK2 NALCN ASTN2 VEGFC REG1B BAGE2 GRIN2B PCDHB4 LRP1B OR2M7 COL19A1 RUNX1T1 GABRG3 ADAMTS20 GABRA2 MXRA5 PTPRT CCKBR PCDH11X OR2L13 SETBP1 COL25A1 NRXN1 FZD10 NTRK3 KEAP1 REG3G C7 GABRA6 OR2T11 OR5W2 ACTN2 OR5F1 ZNF521 OR2T34 LPHN3 DPP6 HEATR7B2 ADAMTS12 OR4A16 ZNF831 OR2T4 OR2G6 MAGEC2 OR4A5 COL3A1 OR8U1 OR2M5 OR10Z1 ASXL3 PGK2 AFF2 NCKAP5 PTPRD SPRYD5 KIF2B INHBA PLCB1 POSTN CDH22 CDH9 SLC17A6 CD5L NETO1 THSD7A OR5L2 SERPINB4 ANKRD30B OR10G8 FLG2 PXDNL SLITRK3 BAGE3 KCNJ12 PCDH10 GRID2 LRRC7 TNN KCNJ3 CNTNAP2 KCNU1 OR8K5 OR5D18 OR2L8 OR5I1 OR4C15 FAT3 REG1A WSCD2 ITGAX OR2G3 PCDHA3 CHRM2 FBXL7 PCDH17 OR4C6 PEG3 GABRB3 GRM7 PCDHB12 POTEH NAV3 CDH7 MYH8 KIAA1211 SLITRK1 OR2T3 ZNF98 OR8H2 CPS1 TRIML1 TRIM48 OR5T3 NRXN3 RP1L1 ANKRD30A EPHB6 OR4M1 KCNC2 DCDC5 ASTN1 DLGAP2 CHRM3 SBSN TLL1 OR4N2 CPXCR1 MMRN1 GFRAL OR2M3 MAGEB4 COL11A1 CTNNA2 OR10Q1 SLIT2 ZNF804A TRPS1 OR2T6 MARCH11 TMTC1 REG3A DCAF12L1 OR2L3 HGF SI ACSM2B PRSS1
EGFR-mut	727	*C11orf16 BCL2L12 KIF26B PSTPIP1 TMEM130 BBOX1 ATRX SLC5A11 AIFM1 SEC24D GSTA1 CHRDL1 LAMA2 PRDM14 NTRK1 DDB1 GAL3ST3 GNAL CRNKL1 RASSF7 ODZ2 SETD2 C15orf48 ARL2 CHD9 USF2 BAK1 UGT2B17 ESX1 GALK1 FABP7 UNC5B MAX IRF2BP2 EPDR1 KIAA1199 TRIM22 CDH13 LAX1 H1FOO HERC2 UNC45B ZSCAN30 CROCC LPAR4 RUNX3 TBL3 WFDC5 UCMA SEC14L5 NFATC1 CCDC106 A1CF UAP1L1 FBXO27 ZFP14 ZNF212 SPAG11B CD1A APCDD1L NAPG YIPF4 DGKH ZNF581 CCDC39 PLP2 ANKRD6 ANO5 OR51Q1 RASL10B VWA5A HIST3H3 NPPB CNOT4 NEFH PCDHA4 MSMB UBQLN4 HIST1H2AE MUC16 GYPC FLT3LG BCL2L10 DAPK3 OR4A47 MARK1 PDE6C SEMA3A COQ9 HYOU1 SORT1 MAP3K13 KLHL30 PCDHGA7 PXDN CENPH RAB35 TOX3 TDRD5 TLR6 RNF215 RCSD1 C19orf55 ZNF334 ESYT3 HIST1H2BC DDHD2 AKR1B15 NR0B1 GDI1 GPRASP2 N4BP2 FCRL3 PCDHGA1 POTEE H3F3B PTPRB LAMB4 GPR52 IZUMO2 GLIS2 SIPA1L2 IGFL4 CDX2 PLEKHG2 TKTL1 UBN2 COL7A1 HIST1H2BJ CHGA HPS4 CRP RAPGEFL1 CALCA KRTAP17-1 TRPC7 KRTAP19-6 TP53INP2 PPIG CRHR2 HEPHL1 HOXD13 IL1RL1 RASGEF1C GPR64 HBG2 LILRA6 PHF1 C12orf59 SMTNL2 AKR7A3 STON1 RPL4 PDRG1 EMR1 PCLO FAIM3 SH3PXD2A PCDHGC5 NR5A1 KLF4 LMO4 MYOD1 GAA ASAP3 RPL10 METTL21D NLRP13 MRPS18A GPR119 CHCHD7 PNLIP PVRIG CDHR1 LRRC66 HECW2 GPBP1L1 CDKN2D CARD14 C11orf71 CCDC120 MTG1 MYBPC3 NPTX2 CRABP1 PCDHGB4 RNF113A RPUSD1 SYTL4 ADRB1 FOXL1 KRT24 MFSD2A TNKS2 RP1 GATS TIFAB TTC3 MS4A13 LYST DDX26B COL23A1 STOML3 PLEKHG3 MYH3 PREX2 UNC13D SIRPB1 LARP1 RIMBP2 MPO PRMT8 PDGFB CDC42BPG UCN2 PCDHGC4 UQCRB TLR8 RNF112 OR1J2 HTR1E MAGIX IRF7 HOXA10 BAI1 ANGEL1 LMO2 HSPA2 PCDHGA10 TNNI2 RPL22 HIST1H3E NUP85 ADAMTS14 HR DBX1 LIN7A PLIN3 RPS26 FTSJD1 TNK2 MATN3 ZDHHC22 EMR2 NPAS4 FBXO17 EIF5A C10orf53 PLEKHA7 ARMCX5-GPRASP2 CTNNB1 EME2 PLD5 WNT9A PCDHGA5 DPPA3 SLC6A13 C1orf52 SFMBT2 LHX8 EAF1 EIF4E1B TRPM2 OPRL1 SPRR4 MYO18A TIE1 EDF1 HIST1H3G SLCO1B1 ITM2C TINAG AXDND1 GCH1 PCDHGA9 MAP7D2 DENND2A MAN2A1 IBA57 NDOR1 EDEM2 BSX MAP2K4 OR5H6 SMAD4 DEFB121 PPP1R14D MAS1L PAK1IP1 GNL1 PPP6R2 CREBZF PCDHGA12 TTC7A PBOV1 FAM127A DNAJB6 CHST2 LHPP AGBL1 KIAA1841 HIST1H2AG CUX2 RPLP2 NLRP9 E2F7 DDX4 SPAG4 CEP89 AP3B2 ZDBF2 TNFRSF12A PCDHGA4 TSPAN14 PPP1R17 CCL2 BEND4 USP51 ADCY10 C17orf56 CEP95 HIST1H4K MS4A6E KIAA0556 COLEC12 PRICKLE2 C3orf27 GPR26 PRRX2 GLRA3 METAP1 WFDC9 XCL2 LTB4R GAPT TMEM89 TDP1 FPR1 PCDHB6 PLIN1 POT1 EIF4B NCKAP1 GZF1 SSPO MAGEH1 DSG4 MLX KDM4A GSG1L ITGAM PGM2 GLYATL1 KIAA1045 SLCO4A1 CNTN3 HIST1H4B ENG ATP6V1F CCDC94 ZBTB37 TMBIM4 NUDT2 C8orf85 NRN1L BRI3BP UHRF1BP1L C9orf142 PRDM8 PPP1R13L OBSL1 IL1RAPL2 FBXO21 STARD8 FASTKD3 SHCBP1 EIF4E COTL1 CLDN15 U2AF2 TMEM155 RMND5A F2RL2 SLC10A1 SLC22A23 LCE1A CDYL2 PLK2 SCARA5 IRX6 LONRF3 PTGDS PCDHGB7 UROC1 FOXC2 CABP4 CTNNA1 NAP1L3 ELK1 GPR20 ZNF428 CCDC3 CPSF1 WBP5 SERPINA7 WDFY3 GRIK1 B3GNT4 GADD45A MSH4 CHTF8 FOXO4 HOXD12 OPN5 INA C1orf50 KRTAP9-8 MOGAT3 SERPINI1 MS4A4A NLN ZSWIM2 GSPT2 SUSD3 CYB5B FHOD1 ODZ1 C7orf59 LHX3 MGA GPR4 CYP4F2 UNC5C BCR SLAMF9 PIK3CA TRAPPC1 FLT3 PCDH19 GALP ABCB9 C16orf90 SRD5A1 RIOK2 CFL2 RASA2 SAP18 AGPAT2 CHRNA4 CALML3 C20orf79 NAT16 ENSA TCEB1 TMEM43 RPL34 KIRREL2 FXYD7 ETHE1 FAM71E1 GPR34 TMC5 WDR77 PPP2R1A NOX3 HSPA12B CEP76 ZDHHC5 ST8SIA3 ZNF880 PROKR2 HNRNPAB ZNF14 CLPSL1 GYPB ZNF426 WNT11 SPTB WDR72 C11orf80 PKD1L1 PCDHGB5 NCOR1 MOCOS BMP1 MBTD1 C16orf72 RGS13 FOXF1 CYBB PCDHGA8 CDK7 PC FOXB2 PDZRN4 KIF1C CCL1 NEUROG2 TMEM105 MFAP2 AP3D1 ABHD12B BIRC8 NIPAL3 DCAF13 MCC SIGLEC10 CPLX2 RB1 STOML2 HTATSF1 BAP1 PDE10A*

		HOXA9 TP53I11 SH2D1B FGD5 ZNF720 CRMP1 FCRL5 ZNF99 CCDC87 OLFML2B SSTR1 C1orf35 NLRP4 ZCCHC8 RAD17 RABEPK CXorf36 FAM133A PDE4DIP IRGC UBA2 UBE2Z TCF3 GDPD2 EIF2AK1 FBLN7 ZNF587 EXTL1 FAM211B TSLP ACVR1B CCDC15 PCDHGA11 PIDD OTUB1 CD320 OR4K5 FAM124A PURB PDK1 DEFB112 PTCHD3 HNF1B BEST2 CXorf40B TNFAIP2 ACN9 RASAL2 SARDH SOHLH1 NOG SH2D4B CD8B CAMKV CLEC5A CANX PCDHGB6 SLC34A2 OR4C11 PTPRJ MLNR KRT25 SNRPD3 MYO1G FOXH1 ATP5L2 ZNF135 TBC1D2 KIF20A FAM104B HPX SMARCAD1 LCE2C STX1B NFXL1 SNX12 MYH4 PRR12 OR2T10 PCDHGC3 FAM65C TIMP2 F2RL3 ZNF789 GTF2IRD1 OCM TUBGCP6 VGF KRTAP9-4 IGF2R ATP8B3 PMS1 DDX39B C17orf39 HDGF GSK3A IGFBP3 TXN2 AGAP1 TNFSF9 ALKBH6 RAB18 C1orf187 HIST2H2AB TMPRSS12 TPH2 NBPF14 CCDC92 DCTN5 ASCL1 CLEC4F TTPA FTCD ERRFI1 NUP43 PTCHD1 AHNAK2 PRAMEF12 RAG2 TSFM PMM2 CIB4 LCE2A BPI LRCH3 MAGED2 CASP2 CAPN7 ZNF341 TESC MAGED1 CSF1R LCE3C PCNXL2 NRK MYL2 SEPT6 CCDC73 OR1S2 PCDHGA6 HIST1H3F GATM CYFIP2 TMEM168 MAP3K11 AGTR2 DNAI2 ZKSCAN2 CWF19L2 FKBP6 SYT16 CCDC58 C19orf21 FXYD3 SCML4 FABP9 SSTR4 FOSL1 PRKCA PCDHGB1 SPANXN3 USH1C DNAH5 UGT1A5 ZNF503 MYBPHL HSPA12A PDE4A NPTXR RNF6 S100A13 SDHA GRIA2 GATA4 CSF2RB ELMOD1 VGLL4 MAP3K9 THNSL2 FCHSD1 HLX TMEM54 SLC6A17 GNL2 ST6GALNAC1 SPOCD1 TRIM50 CDHR2
KRAS-mut	193	MMP2 HPSE2 KCNJ2 ADAMTS18 MAGEA10-MAGEA5 OR13C2 NKX2-5 HS3ST3A1 OR52J3 CABS1 MBL2 UGT3A1 DUSP22 OR10X1 AKR1B10 OR8D4 OR4D9 PRR16 OR10A2 SELL DRD5 KLK8 ROR2 LILRB4 GLRA2 SLC6A15 SYT13 DMRT3 GDF10 SLC8A3 OR7D4 SAMSN1 TMPRSS11E ADAM19 ZSCAN4 DEFB119 GPR148 EN1 NLRP10 KRTAP24-1 OR4D10 COL21A1 RHOH HOXB3 IGSF11 ACTC1 APOB OR4C13 KRAS GIP GRIK4 MYH13 YIPF7 PSG4 VN1R4 ZNF711 MS4A6A MAGEB16 ZCCHC12 CAPN6 SCN3A WT1 ZNF679 HRH2 CETN1 TSPYL2 GABRB1 LILRA2 CAMK2B OR2H1 CFC1B EDN3 LMBRD2 OR2A5 PI15 PRB2 PDILT LOC729264 FCRL2 CFC1 ATM MAPK7 SGCZ OR51G2 MAGEA5 KIR3DL1 ESRRB OTOF U2AF1 DRP2 GLI3 ZIC3 SPERT LMX1A FMO1 LCE1E SULT4A1 OR5M3 SLITRK6 MTNR1A OR2D2 OR13F1 CRISP3 ZSCAN1 CPZ RIPPLY2 CACNA2D3 L1CAM ARMC3 SLC27A6 ZNF835 EDIL3 OR56A3 LSP1 CRX KCNS2 KLK7 DGKI ITGAD PSG2 C20orf85 BCORL1 ZSCAN5B OR14I1 OR2L2 SLC6A5 HIST1H4D CDX4 GIMAP4 TGM6 KCNQ2 KRT38 NEU4 LRRC30 CD163L1 SLC30A10 MAGEA6 TAS1R2 OR11H1 DLX5 CALCR SNRPN TBX3 TBX18 MYH7 TTC29 DLGAP3 CXorf59 GALNT14 SLC9A4 GJC2 MKRN3 PLG IQCJ SEZ6L UBE2A ROBO2 OR52R1 ARHGEF6 RBMXL2 KLHL34 PSG6 OR51A4 GPX5 TEX13A FRMPD1 TCTEX1D1 OR6N1 ARHGAP36 MASP1 SPRR2G HAUS7 LYZL2 KRTAP21-1 TPSD1 GPA33 PARD3B CD300E OR51B5 HS3ST4 TIMD4 OR51I2 OR2Z1 OR52N4 GABRA3 COL1A2 SERPINA5 ST8SIA6 TUBB4A PCDHA6 UGT2B28 NDN MAGEA4
WT	225	USP29 RYR3 CPXM2 NID1 PRKCG MSC ITIH5 C12orf12 PAX4 BASP1 PRKCB ARPP21 FAM5B MAGEB18 DACH1 DAB1 OR8J1 BCLAF1 SYT10 GIMAP1 ZNF648 DBX2 MUC7 OR13G1 TAS2R41 ZIM2 SLC17A3 OR2C3 TUBA3C LTBP1 OR4C16 PCDH18 CYP11B2 CDH2 NCAM1 FSCB CDH8 UGT2B4 MYH2 TSHZ2 MGAT5B ABCB1 OR56A4 KIF19 OR2T27 KCNK10 SLC35F1 OR6N2 SNTG2 NTM JPH2 OR10T2 UNC79 KRTAP6-2 DRD2 COL5A2 PSKH2 GH2 EPHA7 RGS7 SIRPG TTBK1 OR8K1 TFAP2D GPR141 OR10J1 OTUD7A OR2M2 TRPA1 GPR174 NF1 HOXA5 FAM83C DGKB DPYS FSHR LRFN2 TAF1L PASD1 WDR49 WBSCR17 CMKLR1 SLC44A5 HHIPL2 GRM1 GAP43 PDE3A OR10K2 TRHDE OR6Y1 WDR64 CDH11 FHL5 LRRTM1 DCAF8L1 NELL2 SLC10A2 TBX15 PPP1R3A DARC ADCYAP1R1 OR10R2 EPHB1 FAM75A6 GABRA1 LSAMP UGT2A1 RGS1 B3GAT2 LAMP5 PLXNA4 ACSM5 GRIN3A HRNR NPY5R DACH2 MGAM AMPH KDR RGPD4 OR4K1 KCNT2 OR4K13 PCDHB14 F13A1 PCDH11Y TRPC5 OR5B12 OR2A2 LILRB1 PAK7 OR8H1 OLFM4 PIK3CG GUCY1A2 OR6K2 NCAM2 OR4D11 ACSM2A TMEM196 CSMD2 OR8H3 CYLC1 MYT1L SLC12A1 SMARCA4 ASIC2 CNTN1 MEOX2 OR4D6 COL14A1 PRR23A NLGN1 DNAH9 OR5J2 LILRA1 CALN1 ELSPBP1 TPO SEMA6D PCDH8 OR5M10 KCNK13 SEMA3D PTCHD4 ZBBX PKHD1L1 OR2A25 P2RY10 CYP11B1 OR2T8 VSTM2A C6orf118 CYP26B1 OR5AR1 VCAM1 TGIF2LX PCDHB3 UMOD OR51D1 KCTD8 UNC5D TRIML2 GABRG2 FAM190A DAOA NRCAM UGT2B11 UNC13C CES5A FABP12 AGMO NOVA1 SAGE1 ANKRD33 ELTD1 GFRA1 TUBB8 SPHKAP MARCO TBX22 NOBOX RPL10L OR14C36 REM1 GRIA4 TMEM132D C1orf94 C11orf41 ARMC5 FOXF2 OR10A5 KPRP OLFM3 GPR139 SERPINB3 OR4L1 SALL3 NRG1 FAM123C OR5T1 MYO18B FCRL1 DTNA OR4K2

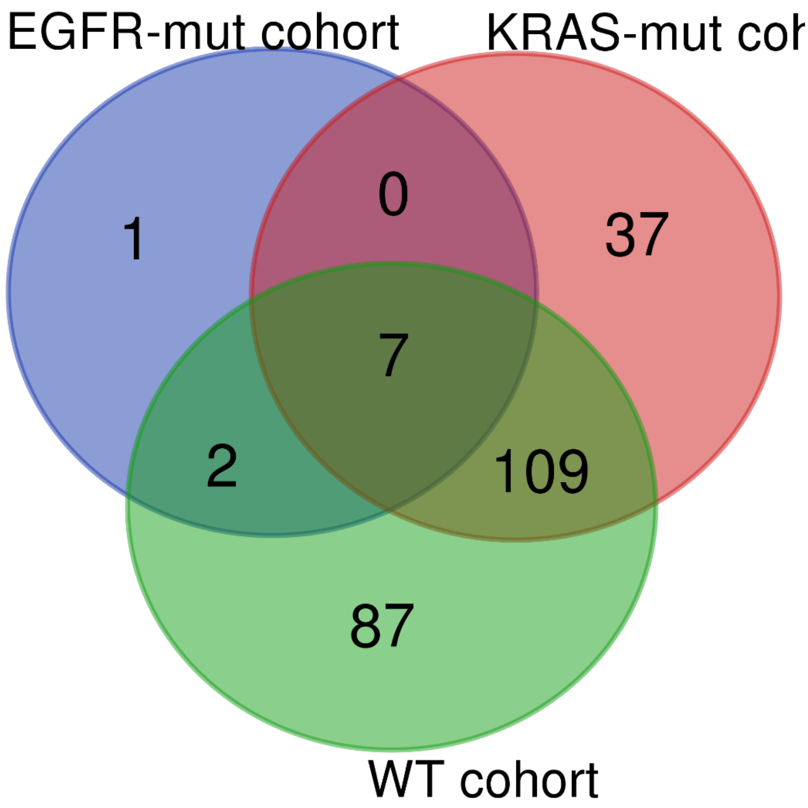

Figure S2. Genes mutated in >10% of members (HPMs) in each cohort excluding EGFR and KRAS in their eponymous cohorts.

Cohorts	Total	Genes
KRAS-mut cohort<br>*EGFR*-mut cohort<br>WT cohort	7	*TP53 CSMD1 AHNAK2 FLG RYR2 PCLO MUC16*
EGFR-mut cohort<br>WT cohort	2	*GRIN2A PKD1L1*
KRAS-mut cohort<br>WT cohort	109	*FLG2 TLR4 RYR3 PEG3 GPR112 PXDNL SCN3A OBSCN CACNA1C TSHZ3 VCAN MXRA5 COL11A1 CUBN CNTNAP5 CDH10 SORCS1 ZFPM2 CTNNA2 LAMA2 CTNND2 ZFHX4 NAV3 COL22A1 PCDH11X ZNF536 FAM75D1 PRUNE2 ASXL3 SVEP1 ZNF804A MYH8 LAMA1 FAM5C DNAH11 SETBP1 FAT4 MAGEC1 HCN1 ODZ3 RELN TRPS1 XIRP2 DOCK2 ANK2 ASPM NALCN NRXN1 HMCN1 NCKAP5 DNAH9 FAM135B C1orf173 KEAP1 PTPRD RYR1 APOB PCDH10 NLRP3 HYDIN FAM47B LRRC7 LRP2 EPHA5 TG CPS1 TNN SYNE1 TNR GRIN2B RIMS2 DMD CRB1 CNTNAP2 COL6A3 RP1L1 LRP1B PKHD1L1 PRDM9 SI NEB CDH9 LPPR4 MYH1 FBN2 LPHN3 ASTN1 THSD7A DST FAT3 CACNA1E TIAM1 AHNAK ODZ1 CDH12 SNTG1 COL19A1 ABCA13 ADAMTS12 ZNF831 NOTCH4 C15orf2 KIAA1109 PAPPA2 PCDH15 SLITRK2 CSMD2 HRNR SSPO*
EGFR-mut cohort	1	*LAMB4*
KRAS-mut cohort	37	*ERBB4 ABCB5 TPTE ZNF479 TRPC4 MMP16 PTPRT ROBO2 OTOF STK11 ACAN NLRP5 3 F3 AFF2 PTPRB F8 NRXN3 GRM8 FCGBP MYH7 KCNU1 ZP4 EPHB6 TRPM6 ZNF521 ADAMTS2 MYH13 MLL2 BCORL1 RBM10 NLRP14 ITGAX NELL1 ANKRD30B OTOGL ATM*
WT cohort	87	*ADAMTS20 MYT1L SPEG GPR98 SORCS3 SCN2A SMARCA4 MGAM COL3A1 C11orf41 DCHS2 DNAH8 KDR RGPD4 SLIT2 EPHA6 GPR158 MLL3 LRRIQ1 MUC5B EPHA3 NID1 ODZ2 KCNT2 CDH18 CDH23 LYST ADAMTS16 SYNE2 COL14A1 UNC79 BAI3 ZNF208 ASTN2 POM121L12 DNAH3 DNAH7 LTBP1 COL5A2 KIF2B LRRTM4 DUSP27 FAT1 GRID2 MYO18B PLEC SPTA1 HERC2 FLNC UNC13C PTPRZ1 FAM47A MYO7B PLCB1 PKHD1 PPP1R3A ANKRD30A WDFY3 ITGA8 COL6A6 FAM47C CDH8 SLC8A1 HGF DNAH5 TRPA1 BIRC6 TEX15 FMN2 NF1 FAM75A6 MYH2 LPA COL12A1 MYH4 HEATR7B2 LCT SPHKAP ZNF804B SEMA5A FER1L6 HECW1 PLXNA4 PCDH17 SPEF2 TAF1L SLC39A12*

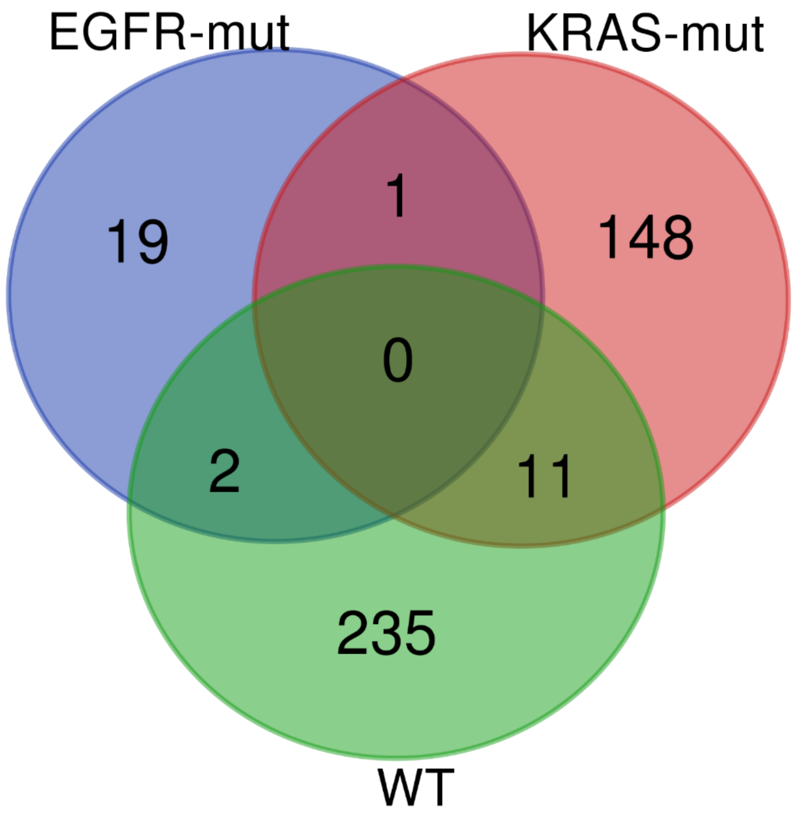

Figure S3. Conserved genes defined as >2Standard Deviations below the neutral line in LUAD cohorts.

Cohorts	Total	Genes
EGFR-mut cohort<br>*KRAS*-mut cohort	1	*REV3L*
EGFR-mut Cohort<br>WT Cohort	2	*HTT VPS13D*
KRAS-mut Cohort<br>WT Cohort	11	*MDN1 FAM75A4 NBPF10 LRRC37A2 LOC100132247 NBPF11 FAM75C2 ZNF729 RGPD6 RIMBP3 CACNA2D2*
EGFR-mut Cohort	19	*RYR3 HERC1 MACF1 FAT4 SACS ZFHX3 MLL2 SPTBN5 CSMD2 TNXB RANBP2 USP34 DYNC1H1 HSPG2 LAMA5 FREM2 BRCA2 C12orf51 DNAH10*
KRAS-mut Cohort	148	*ESPL1 ATP2A1 ANKS1A COBLL1 KIAA1524 PAXIP1 ASAP2 NARG2 IPO11 UBR5 ANKRD36B BRD4 WWC2 IFT122 PIDD COPB1 ERAP2 DENND5A TBC1D2B INF2 IGSF9 SEC16A MED24 VCPIP1 PIKFYVE FAM75A5 BRPF3 DENND4C PHC1 HPS3 ZC3H4 HMHA1 RAB3GAP1 PIGO ZNF827 TMPRSS9 CHERP BRPF1 HR NOL8 SART3 MAML2 ASXL1 USP25 ANO8 DMXL2 MRVI1 RALGAPA1 KIAA0182 FBXW10 BCAR1 PRR12 SEC24B KIAA0753 RBM33 GARNL3 KIAA2018 MARS TNPO1 GCFC1 CAMSAP3 NEFH SLC26A8 SAFB2 WNK4 CDK13 INPP4A AFTPH ATP8B3 JMY AP2B1 CHTF18 POM121C GTF2IRD2B SIPA1 HEATR4 DGKQ MKL1 MEGF11 CLSTN3 NISCH PPP1R10 PDZD8 PHC3 PHF12 NUP153 RBM12B NCOA7 CCP110 MBTPS1 FAM75D3 MAP3K6 PWP2 KIAA0195 ZZEF1 HEATR7A FAM75A7 ATP2A2 NPAT ZNF839 ZNF687 EFTUD2 KIAA0355 PLEKHA6 ZNF845 KDM2A FAM13B NRD1 SCAF4 GTF3C2 GPR113 FAM75D4 EXTL3 EEA1 WDR62 CIITA NBR1 LTBP3 TERT EDEM3 CCDC18 ST5 CLASP2 LRIG2 WAPAL UPF1 SLC24A1 CASP8AP2 USP37 TP53BP2 SPG11 NOMO2 EPHA1 EIF4G1 ARHGEF5 ETAA1 NLRC3 GANC DGKD SPOCD1 SRRM1 DUOX1 GBA2 KCTD19 TMCO7 KIF23 ASAP3 FILIP1L*
WT Cohort	235	*STRC NACC1 CLOCK EIF2B5 CCDC149 GNL3 DDX51 LOC728393 C4B PMPCA FAM65A FES ZNF324 DCAF10 GLUD1 ZNF84 AGFG1 KIAA1217 MNT SLC34A3 SENP1 CCDC157 NBEAL2 CDY2A CRTC3 EPB41 COG4 RMI1 LOC728405 TRIM45 MAGED4 TCP1 CWF19L1 FAM20A TBC1D3H KLC4 KRIT1 MAGED4B FLVCR1 AVIL NEDD9 SNX33 RNF19B LOC100293534 PGM1 AGAP8 GOLGA8A ANKRD20A2 ZFP106 ZNF726 POTED MLLT1 TCEB3CL TMEM180 ENTPD3 LOC728400 ACADVL SGSM2 SLC44A2 EPN3 ANKDD1A KCNS1 KCNC3 HPS6 CDK5RAP1 TNK1 ACBD3 ANKRD11 C14orf45 MYSM1 MPP7 LOC728379 DYNC1LI1 EHD2 TBC1D3C C17orf80 ANKRD6 SHROOM1 CBS TCEB3C FASTK NOA1 POMT2 FXR2 CCNK TOX4 PLEKHG1 EEF1D ZBTB1 RGPD8 SREK1 HEXB SPNS2 IRF2BP1 ZNF749 PPP1R16A NSUN7 UBP1 FAM160B2 CCDC111 COL4A3BP HDAC5*

	SOCS6 PLEKHN1 MEGF9 MFSD5 CDY1B IPO5 SAMD11 ZNF642 DUS3L SLC1A4 FRMD5 AP5B1 KIAA0368 ZNF30 CRTC1 ABCF1 GPT2 SORT1 LOC728373 NDOR1 HOOK2 NEMF IRF5 NTN1 CTU2 CRAT ZDHHC5 SLC19A1 SREBF2 CSRNP1 UBXN11 AKAP9 SACM1L GALNS EHD4 NELF TFR2 DHX33 ICAM1 LRRC37A PRKAG2 WDR26 CDY2B PCDHGB5 REC8 TRAFD1 PIK3R2 ZNF57 ZNF433 MYO5C FAM48B2 ii4B TAP1 RNF123 MBTD1 NT5DC2 UNC45A MAP3K1 CABIN1 ERAP1 SYTL3 KDM4B RGPD5 DDX56 TUBGCP4 MAP4K5 C4A GSPT1 NOMO3 LRRC48 FUT4 AMOTL2 ADCK5 IL1R1 HGS SH2B3 STXBP3 SUZ12 PRRT3 ZNF655 HIF1A SLC7A8 RANBP9 C17orf56 BANP CEP104 CEP95 ZNF503 GGT5 TAF6L SIN3B PVRL2 LOC100506888 LOC728369 TDRKH CDY1 TRIP4 PANX2 AGTPBP1 GTPBP2 HSF1 REPS1 GPR108 IL27RA PDXDC1 ZNF846 STOX2 PPIP5K1 VASN XRN1 AGAP9 FAM48B1 TMEM201 C14orf21 AVL9 ZNF205 TTC30A C2CD2 ZNF26 PITPNM1 PDE12 EIF2AK4 TRIP11 SLC22A5 RNF213 ATL1 ARHGEF1 NBPF24 USP17L5 ACSF2 CAMTA2 SLC44A4 RXRB

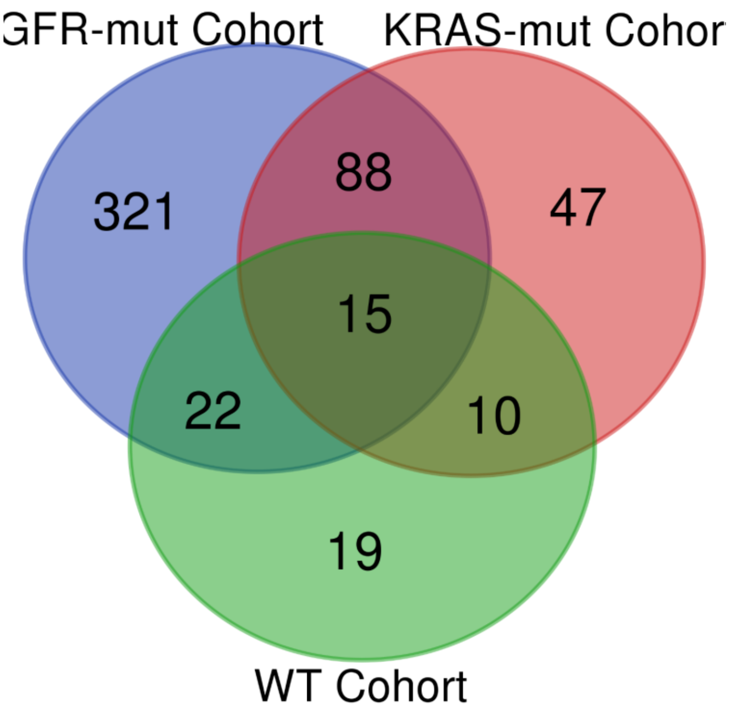

Figure S4. Genes with 0 mutations and > 4-fold increased expression in each cohort.

Cohorts	Total	Genes
EGFR-mut Cohort<br>*KRAS*-mut Cohort<br>WT Cohort	15	*SPINK1 FGF11 MB CCNB2 BARX2 GJB2 S100A2 FDCSP XAGE1B XAGE1D RHOV PRSS3 DDIT4L CDKN3 SLC2A1*
EGFR-mut Cohort<br>*KRAS*-mut Cohort	88	*CCNO CXCL14 GOLGA7B GOLM1 B3GNT3 OCIAD2 CXCL13 TMEM184A ONECUT1 STX1A CDCA3 GINS1 YBX2 C16orf59 ZIC2 HIST1H2AM P2RY6 GJB6 AK4 CXorf61 POLE2 FAM177B ENTPD8 LEMD1 HOXC10 EFNA4 PPAP2C MDK ASF1B PODXL2 MIOX CDC45 SPC25 WDR62 CST1 RAB26 PHLDA2 GPC2 BCL2L15 C14orf105 KRTAP4-1 IL36RN FGL1 IGSF9 EME1 FERMT1 WFDC3 ANKRD22 HOXB9 RTN4RL2 PCP4 TMPRSS4 ETV4 NMU RGS17 HIST1H2BD PYCR1 CST2 CENPA LY6D CBLC TUBB3 KIAA0101 SLC7A10 ONECUT2 IGFL2 TFF1 KDELR3 GAD1 SCG5 PBK KRT81 RHBDL1 SPINK13 CDCA5 KRT86 GINS2 UBE2T FAM64A NUSAP1 RHBG LYPD1 AGR2 RDM1 PROC STEAP1 KIF23 DERL3*
EGFR-mut Cohort<br>WT Cohort	22	*NXPH4 NKAIN1 EPN3 PLEKHN1 FRMD5 PRSS50 C1QTNF6 ALG1L TFR2 APOBEC3B PANX2 PAEP PAFAH1B3 IL23A ITPKA GCNT3 EFNA3 CNFN GPT2 CTHRC1 FUT2 NEK2*
KRAS-mut Cohort<br>WT Cohort	10	*S100P C15orf48 CKMT1B PLEK2 PPP1R14B NQO1 AURKA SLC7A11 HMGA1 NME1*
EGFR-mut Cohort	321	*PNCK ESPL1 SPINK2 FEZF1 CDH3 KLHDC8A PAX7 E2F8 DNASE1 ZNF695 SKA3 SRCIN1 XDH IQGAP3 CGREF1 ANKRD34B GFRA3 TK1 ZPLD1 CHGB MFSD6L VIL1 ADAM12 SLC52A1 PLAU CST4 KLK6 SLC6A11 STYK1 C7orf10 TPX2 CNGB1 DEPDC1B CILP CNNM1 SLC2A5 IGF2BP3 HOXC13 HMGB3 DEPDC7 SLC29A4 CRABP2 SPAG5 KRT80 PROM2 B3GAT1 LY6K FAP VSTM2L BCMO1 B4GALNT4 TRPM8 AKR1B10 CYP2D6 DNAJC22 COL9A2 TCN1 COL1A1 C19orf26 BRIP1 CYP27B1 ANLN FOXP3 BIRC5 PNMA6A CHRNA5 SLC1A7 T PLK1 C1QL2 SLC15A1 TNFSF11 NETO1 IL37 SYT2 CLDN3 PABPC1L GPR115 STIL SCUBE3 MYEOV UBE2C ADAM28 CNGA3 MND1 MEX3A TMEM63C CCNE1 FOXM1 FAM150A SRPX2 HOXC9 BPIFB2 SYT13 C6orf222 CASC5 FUT9 FUT3 CDT1 UGT2B15 FNDC1 SOHLH2 SYT12 UPK3A TONSL KIF11 CEP55 SMC1B TMEM156 SPDEF HPCA TMPRSS11E EPYC KIF18B KRT16 RRM2 PAX9 DLX3 JSRP1 COMP TRIM54 SIX4 SRPK3 C1orf65 FOXB1 ARSH HELLS HHLA2 TNS4 CLDN2 GRIK2 CHST6 BRDT PLAC1 HTR3A CDC25C C10orf81 FUT6 HOXB13 CDC6 GSDMB VTCN1 ECEL1 CLDN6 KCNQ5 PKMYT1*<br>*STRA6 IL31RA MYBL2 SPTBN2 MMP17 HOXD4 KIF15 CEACAM7 WFDC2 LRRC31 OTX1 KRT15 CASR SBK1 TFAP2A BUB1B C17orf53 PTPRH NR2E1 PLXNB3 DLGAP5 GLYATL2 TGM5 PGM2L1 DPEP1 ISX HJURP ABCA4 UNC5CL E2F2 RHBDL2 CDH17 GFAP IL1RL2 MYBPH SERINC2 AURKB IL22RA2 HOXD3 CKAP2L BAI2 CYP27C1 CLDN9 MYO7A PITX2 CABYR SPP2 CLSPN CR2 HOXB8 ABP1 ACMSD HSF2BP NCCRP1 SGOL1 SULF1 SLC16A9 BMP8A RAMP1 CDCA2 EGF B3GNT6 MARCKSL1 HMMR SIX2 KIF4A METTL7B TMEM59L IGSF9B ABCC3 CHAF1B EXO1 HOXC11 DOK5 RHCE STK32A TFAP2D CENPI LGSN MUC21 XKRX ERCC6L KIF2C EDN2 ST8SIA2 MCM10 WDR86 ORC6 GCM1 PPP2R2C EPHX4 MUC13 OVOL1 LINGO1 IL4I1 SGK2 HNF4G SLCO5A1 TEKT5 HOXC8 COL10A1 CHTF18 C5orf34 F12 SYT7 COL24A1 SYNGR4 GPR87 ALB CEACAM1 MELK PPFIA4 HHIPL2 SMPX HOXC6 CRYGN CDC20 IL36G DNAJC12 ZWINT MANEAL PNMA3 DMBX1 ARHGEF19 RECQL4 LRAT SPERT QPCT PGLYRP4 HOXB7 MFI2 SPTSSB CCNA2 LGR4 TTK SULT4A1 HAVCR1 GTSE1 EPHB2 POU3F2 BAIAP2L2 CKM COCH NCAPG RCC1 ACHE KRT6A GNGT1 DUSP9 TBX15 CYP24A1 PVRL4 SKA1 CPNE7 FADS6 HRASLS GYLTL1B C8orf73 ENPP3 HABP2 CASKIN1 GPR110 HMGA2 CAMK2N2 SLC6A3 ALPK2 ACY3 LAMP5 MMP9 DEPDC1 SPOCK1 EEF1A2 FAM83A XRCC2*
KRAS-mut Cohort	47	*C1orf106 DIRAS1 BPIFA2 B3GNT4 HIST1H2BG APCDD1L SLC7A5 NKAIN4 PPAPDC1A DUSP4 MSMB PRR19 DBNDD1 SPINK4 TNFRSF21 WNT3 TERT GJB3 AKR7A3 IL17C COX6B2 FGF19 RCOR2 FHL2 HOXA10 C12orf56 HS6ST2 INHA LYPD3 SIX1 FAM72D CLDN10 CDCA7 ASPHD1 GREM1 TESC OIP5 FBXO32 TRIM15 KCNN4 HORMAD1 PRR11 IBSP RNF183 SPRR1B*
WT Cohort	19	*CCNB1 MAGEA2 RAB3B FAM72B VPREB3 GPR19 FAM72A RAC3 RNF186 C9orf100 C5orf46 GNG4 CDK5R2 IGFBP3 SPAG4 GAL C1orf135 CENPM RPL39L*

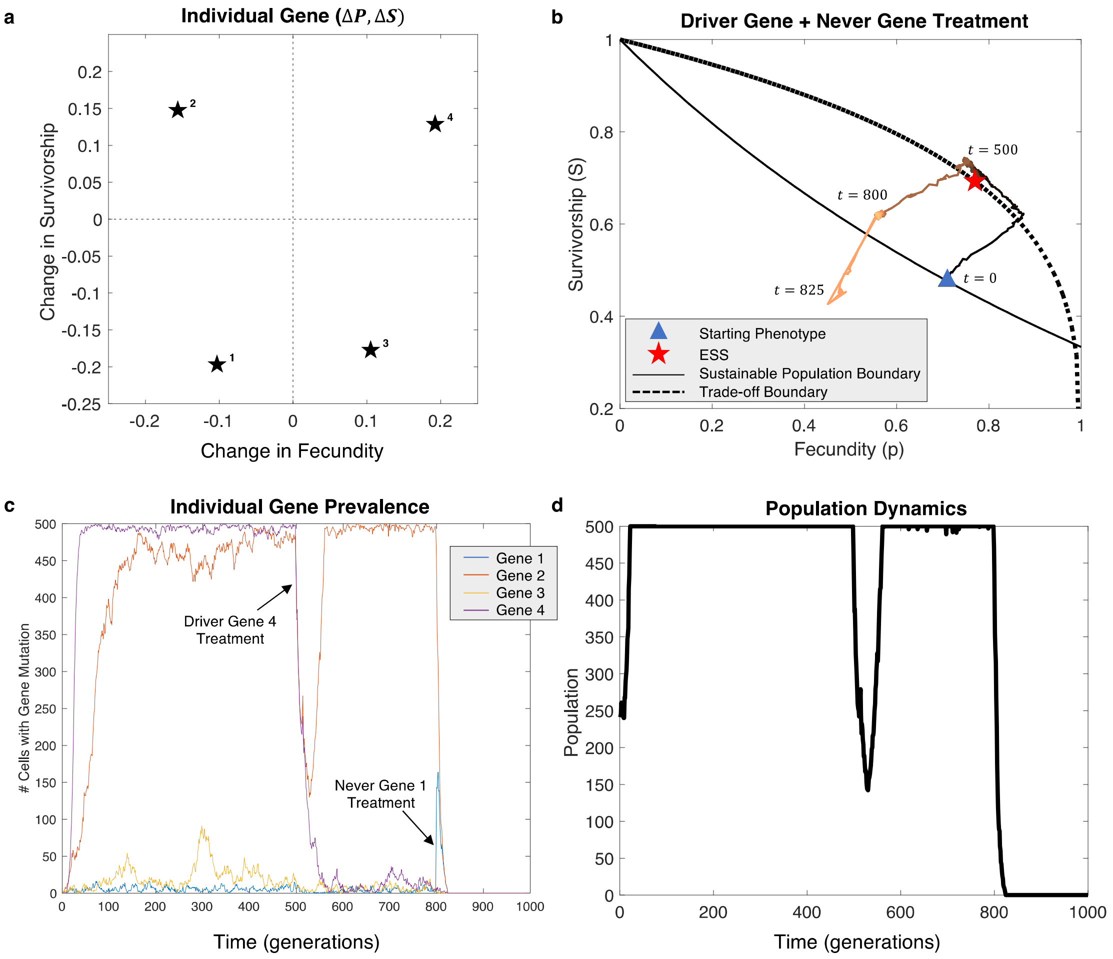

Figure S5. (Ref (1)). Panel (**a**) shows four simulated gene mutations that confer unique changes to fecundity and survivorship of a particular cell. For example, a mutation in gene 4 increases both fecundity and survivorship while a mutation in gene 2 increases survivorship but decreases fecundity. Panel (**b**) shows the simulation space with a sustainable population boundary (solid line) where population size does not change. This is where normal cells are found, and the starting phenotype of cells simulated here is highlighted with a blue triangle. In cancer, cells evolve from their original phenotype towards to the dotted line which represents a trade-off between survivorship and fecundity, above which cells begin to require too many resources and are unable to survive. The point at which fitness in maximized, known as an evolutionary stable strategy (ESS), is highlighted with a red star. Panel (**c**) shows the mutation prevalence over time for the four genes. The population acquires a mutation in gene 4 followed by mutation in gene 2 providing an evolutionary trajectory (shown in panel **b**) approaching the ESS. Here we consider gene 4 to be the driver mutation and a targeted therapy is applied at time = 500. The prevalence of cells with a mutation in gene 4 plummets, as does the population density (shown in panel **d**). As with many targeted therapies though, the population can recover due to other mutations within the population, even if the resulting fitness is relatively lower (as shown in panel **b** at time = 800). Interestingly, gene 1 is conserved (never mutated) in this cell population, as it confers decreases in both fecundity and survivorship. However, if a therapy is then used to disrupt this conserved gene, the fitness of the cell population falls well below the sustainability line, and the population collapses. Please see (Ref (1)) for a more detailed analysis.

Supplementary Tables

Table S1. The most frequently mutated genes in each cohort along with the number of samples with this mutation. The observed number of mutations is compared to the number expected by chance alone based on the gene size and the mutation rate through robust regression analysis (see Methods). The distance from the neutral line was measured for all genes and is presented as a standard deviation from the mean distance. Higher standard deviations represent the most likely gene mutation under positive selection. Note some large genes are among the most frequently mutated but still not likely to be under evolutionary selection.

Mutational frequency								
EGFR-mut N=58			*KRAS*-mut N=163			WT N=313		
Gene	Samples mutated	Distance from neutral line (standard deviation)	Gene	Samples mutated	Distance above neutral line (standard deviation)	Gene	Samples mutated	Distance from neutral line (standard deviations)
TP53	34	12.1	*MUC16*	79	1.8	*TP53*	177	11.9
MUC16	12	2.7	*LRP1B*	66	5.0	*CSMD3*	135	6.2
CSMD3	11	4.9	*RYR2*	66	5.4	*MUC16*	132	3.3
RYR2	10	4.6	*CSMD3*	65	5.5	*RYR2*	125	5.6
MUC17	9	4.0	*ZFHX4*	60	5.8	*LRP1B*	117	4.7
CSMD1	7	4.4	*TP53*	57	8.2	*USH2A*	105	3.7
AHNAK2	6	2.5	*USH2A*	54	3.7	*ZFHX4*	104	5.5
FLG	6	2.9	*CSMD1*	46	4.9	*XIRP2*	101	4.7
GRIN2A	6	4.0	*XIRP2*	43	3.8	*FLG*	93	3.9
LAMB4	6	3.9	*FLG*	39	3.0	*SPTA1*	88	5.0
PCLO	6	2.7	*SPTA1*	39	4.2	*COL11A1*	82	5.4
PKD1L1	6	3.4	*ANK2*	37	2.9	*NAV3*	75	4.4
SPTA1	6	3.6	*ZNF536*	37	5.2	*FAT3*	74	2.6
USH2A	6	2.6	*RP1L1*	35	3.9	*ZNF536*	69	5.4
ATRX	5	3.1	*APOB*	34	2.2	*PCDH15*	68	4.4
CACNA1E	5	3.4	*FAT3*	33	2.2	*MUC17*	67	2.1
CRB1	5	3.6	*PAPPA2*	32	4.0	*RYR3*	66	1.8
DNAH5	5	2.3	*STK11*	32	7.7	*PCLO*	66	2.2
HERC2	5	2.3	*CNTNAP2*	31	4.5	*NRXN1*	65	3.7
HMCN1	5	2.0	*COL11A1*	31	3.9	*CSMD1*	65	2.1
MGA	5	3.0	*KEAP1*	31	5.6	*DNAH9*	65	5.1
ODZ1	5	3.0	*LRRC7*	31	4.2	*ZNF804A*	63	2.3
PAPPA2	5	3.4	*GPR112*	30	2.7	*TNR*	63	4.2
TNR	5	3.6	*NAV3*	30	3.3	*SI*	63	4.8
WDFY3	5	2.7	*PCLO*	30	1.6	*ANK2*	63	5.0
XIRP2	5	2.7	*PXDNL*	29	4.6	*ADAMTS12*	62	4.4
			RYR1	29	1.8	*GPR112*	60	2.8
			RYR3	29	1.8	*KEAP1*	59	1.5
			ADAMTS12	28	3.8	*CDH10*	59	5.4
			DST	28	1.4	*APOB*	59	6.1
			PCDH11X	28	4.1	*PAPPA2*	58	1.1
			RIMS2	28	4.0	*FAT4*	58	0.7
			CACNA1E	27	3.3	*HMCN1*	58	3.9
			MUC17	27	1.5	*PTPRD*	57	1.5
			NALCN	27	3.5	*FBN2*	57	2.8
			OBSCN	27	0.6	*PKHD1L1*	57	2.2
			C15orf2	26	4.0	*ABCA13*	57	3.7
			FAT4	26	1.1	*CSMD2*	56	2.2
			MXRA5	26	2.6	*RYR1*	56	1.5
			NRXN1	26	3.9	*C1orf173*	55	4.1
			ATM	25	2.2	*RP1L1*	55	3.2
			DMD	25	1.8	*FAM135B*	54	4.1
			DNAH9	25	1.3	*ASTN1*	52	4.2
			FAM135B	25	3.6	*PKHD1*	52	1.4
			FBN2	25	2.3	*CACNA1E*	51	0.2
			HMCN1	25	0.6	*FLG2*	51	3.2
			PTPRD	25	3.1	*RELN*	51	2.8
			TSHZ3	25	4.0	*AHNAK2*	51	0.1

	COL22A1	24	3.6	*OBSCN*	51	1.8
	PRDM9	24	4.1	*SORCS1*	50	4.2
	RELN	24	1.8	*C15orf2*	50	1.6
	SNTG1	24	4.7	*PCDH11X*	50	2.4
	AFF2	23	3.4	*HRNR*	50	0.8
	ASXL3	23	2.8	*DMD*	50	3.9
	C1orf173	23	3.3	*LRP2*	50	4.4
	CDH10	23	4.1	*PEG3*	49	0.6
	EPHA5	23	3.9	*NALCN*	49	3.3
	PCDH15	23	2.9	*NF1*	49	2.3
	SVEP1	23	1.9	*VCAN*	49	3.5
	VCAN	23	1.7	*MLL3*	49	-1.8
	ZNF831	23	3.3	*SYNE1*	49	1.7
	ABCA13	22	0.9	*PRDM9*	47	-0.7
	ASPM	22	1.5	*TNN*	47	4.3
	COL6A3	22	1.7	*UNC79*	47	3.9
	FAM5C	22	4.0	*NEB*	47	2.4
	MYH8	22	2.7	*CNTNAP5*	46	3.4
	SI	22	2.8	*BAI3*	46	4.0
	SSPO	22	1.7	*LRRC7*	46	0.6
	SYNE1	22	-1.3	*DNAH5*	46	0.3
	TPTE	22	4.3	*DST*	46	3.3
	ABCB5	21	3.4	*MXRA5*	45	1.3
	CNTNAP5	21	3.5	*ASPM*	45	0.9
	DOCK2	21	2.7	*DNAH11*	45	2.1
	ERBB4	21	3.2	*PXDNL*	44	3.3
	FLG2	21	2.2	*COL22A1*	44	3.6
	KIAA1109	21	0.4	*ADAMTS20*	44	0.3
	LAMA2	21	1.6	*ZNF831*	44	2.7
	NCKAP5	21	2.7	*MYH1*	44	4.0
	NLRP3	21	3.5	*DNAH8*	44	3.3
	PRUNE2	21	1.7	*PCDH10*	43	-0.6
	AHNAK	20	-0.2	*COL3A1*	43	3.6
	ASTN1	20	3.1	*ODZ1*	43	1.8
	CPS1	20	2.8	*TG*	43	3.8
	CUBN	20	1.1	*AHNAK*	43	1.8
	FAM47B	20	3.9	*EPHA5*	42	3.8
	MYH1	20	2.4	*PCDH17*	42	2.6
	NLRP5	20	3.4	*MYH2*	42	3.6
	SCN3A	20	2.4	*SNTG1*	41	2.4
	SETBP1	20	3.0	*TSHZ3*	41	3.2
	HCN1	19	3.7	*KCNT2*	41	1.3
	HRNR	19	1.6	*COL5A2*	41	3.6
	MYH7	19	2.3	*TAF1L*	41	4.6
	NEB	19	-0.8	*ASXL3*	41	2.6
	OTOGL	19	2.7	*COL6A3*	41	3.6
	PKHD1L1	19	0.9	*CDH9*	40	4.0
	PTPRT	19	2.8	*LPHN3*	40	0.5
	ROBO2	19	2.8	*FMN2*	40	3.3
	TG	19	1.6	*SPHKAP*	40	3.8
	TNN	19	3.1	*PLEC*	40	1.0
	TRPS1	19	2.9	*DNAH3*	40	2.7
	ZFPM2	19	3.1	*HCN1*	39	3.1
	ZP4	19	4.0	*FAM5C*	39	3.9
	BCORL1	18	2.5	*CDH12*	39	3.5
	CTNNA2	18	3.2	*CTNND2*	39	3.9
	EPHB6	18	3.1	*ANKRD30A*	39	0.0
	FAM75D1	18	2.5	*PLXNA4*	39	4.0
	FCGBP	18	-0.1	*PRUNE2*	39	1.3
	GRIN2B	18	2.6	*LAMA1*	39	1.2
	HYDIN	18	-0.1	*LAMA2*	39	2.5
	LPHN3	18	3.2	*FAT1*	39	1.3
	NRXN3	18	3.0	*FAM47A*	38	2.4

	OTOF	18	2.2	*CTNNA2*	38	3.6
	PCDH10	18	3.1	*FAM47C*	38	2.4
	SLITRK2	18	3.3	*LTBP1*	38	3.8
	THSD7A	18	2.6	*NCKAP5*	38	3.4
	TLR4	18	3.3	*COL14A1*	38	-0.4
	TNR	18	2.7	*DOCK2*	38	2.8
	ZNF804A	18	2.9	*SSPO*	38	-0.1
	ADAMTS2	17	3.2	*MUC5B*	38	2.5
	AHNAK2	17	-0.6	*HYDIN*	38	1.0
	ANKRD30B	17	4.4	*SYNE2*	38	-1.6
	CDH9	17	3.3	*CDH18*	37	3.2
	CRB1	17	2.5	*TLR4*	37	3.7
	GLI3	17	2.4	*ADAMTS16*	37	1.1
	KCNU1	17	3.0	*RIMS2*	37	2.7
	LAMA1	17	1.1	*CRB1*	37	2.8
	MAGEC1	17	2.8	*CPS1*	37	0.6
	NLRP14	17	2.9	*FAM75D1*	37	2.7
	ODZ1	17	1.3	*MGAM*	37	-0.5
	ODZ3	17	1.6	*ODZ2*	37	2.4
	PEG3	17	2.3	*COL12A1*	37	1.9
	TRPC4	17	3.0	*CUBN*	37	2.9
	ZNF521	17	2.6	*KIAA1109*	37	3.6
	ACAN	16	1.6	*KIF2B*	36	3.2
	CACNA1C	16	2.0	*SLITRK2*	36	2.9
	CDH12	16	3.1	*DUSP27*	36	3.2
	COL19A1	16	2.7	*SLC8A1*	36	0.2
	CSMD2	16	0.7	*ASTN2*	36	3.4
	CTNND2	16	2.8	*COL19A1*	36	2.8
	DNAH11	16	0.2	*GPR158*	36	3.1
	F8	16	1.5	*ZFPM2*	36	2.5
	GRM8	16	2.9	*TRPA1*	36	3.8
	ITGAX	16	2.8	*PPP1R3A*	36	2.1
	LPPR4	16	3.4	*CNTNAP2*	36	2.3
	LRP2	16	-0.1	*FAM75A6*	36	1.2
	MLL2	16	-0.2	*SETBP1*	36	3.1
	MMP16	16	3.4	*HECW1*	36	2.7
	MYH13	16	2.0	*MYO18B*	36	3.3
	NELL1	16	3.1	*SPEF2*	36	3.5
	NOTCH4	16	1.9	*MYH8*	36	2.2
	PTPRB	16	1.7	*PKD1L1*	36	3.1
	RBM10	16	3.7	*DNAH7*	36	3.1
	SORCS1	16	2.8	*POM121L12*	35	1.9
	TIAM1	16	2.2	*LPPR4*	35	3.9
	TRPM6	16	1.8	*NLRP3*	35	3.2
	ZNF479	16	3.5	*SMARCA4*	35	1.5
	ADAMTS20	15	2.1	*ZNF208*	35	4.7
	ADAMTSL3	15	1.9	*TRPS1*	35	1.6
	BAI3	15	2.1	*TIAM1*	35	2.9
	BOD1L	15	1.1	*UNC13C*	35	2.4
	C12orf51	15	0.4	*COL6A6*	35	2.8
	CD163L1	15	2.1	*PTPRZ1*	35	2.3
	CHL1	15	2.4	*ODZ3*	35	0.5
	COL12A1	15	0.8	*WDFY3*	35	2.8
	COL3A1	15	2.4	*SLC39A12*	34	-0.2
	DNAH7	15	0.1	*HGF*	34	1.3
	DNAH8	15	-0.3	*SEMA5A*	34	2.7
	FAM47C	15	2.6	*MAGEC1*	34	3.7
	FRMPD1	15	2.0	*PLCB1*	34	0.2
	GRID2	15	2.7	*HEATR7B2*	34	2.9
	HEATR7B2	15	2.3	*FLNC*	34	2.8
	HEPH	15	2.7	*TEX15*	34	3.0
	KIF2B	15	3.1	*LYST*	34	3.8
	KLHL1	15	3.0	*BIRC6*	34	1.0

	LPA	15	1.6	*LRRTM4*	33	2.1
	LRFN5	15	3.1	*FAM47B*	33	3.3
	MED12	15	1.8	*CDH8*	33	0.9
	MYH2	15	1.7	*EPHA6*	33	3.2
	NTRK3	15	3.0	*GRIN2B*	33	3.6
	ODZ2	15	1.4	*CACNA1C*	33	2.3
	OR2L2	15	3.8	*RGPD4*	33	3.8
	OR2W3	15	3.8	*MYH4*	33	1.8
	OVCH1	15	2.8	*DCHS2*	33	2.0
	PCDH17	15	2.5	*SVEP1*	33	0.7
	PCDHA2	15	2.7	*EPHA3*	32	2.0
	PKHD1	15	0.1	*ITGA8*	32	3.0
	RBFOX1	15	3.6	*GRID2*	32	1.8
	SLIT2	15	2.1	*GRIN2A*	32	2.9
	SLITRK1	15	3.1	*THSD7A*	32	2.3
	SORCS3	15	2.8	*SLIT2*	32	2.9
	SYNE2	15	-1.5	*C11orf41*	32	1.7
	TAS1R2	15	2.9	*NOTCH4*	32	1.8
	TRRAP	15	0.2	*FER1L6*	32	2.2
				LCT	32	1.6
				SPEG	32	2.2
				MYT1L	31	0.7
				SORCS3	31	-1.5
				NID1	31	-0.8
				ZNF804B	31	2.3
				KDR	31	1.5
				MYO7B	31	1.9
				LRRIQ1	31	2.3
				SCN2A	31	3.3
				SCN3A	31	2.7
				LPA	31	1.6
				CDH23	31	1.6
				HERC2	31	3.0
				GPR98	31	2.3
				OR4A15	30	11.9
				SLITRK1	30	6.2
				NLGN4X	30	0.3
				UNC5D	30	5.6
				GRM8	30	4.7
				CNTN5	30	3.7
				GRM1	30	5.5
				ATRNL1	30	4.7
				ZNF521	30	3.9
				AFF2	30	5.0
				SALL1	30	5.4
				PTPRT	30	4.4
				MYH6	30	2.6
				DIDO1	30	5.4
				PDE4DIP	30	4.4
				STAB2	30	2.1

Table S2. 7 of 10 genes mutated in >10% of the *EGFR*-mut tumors are also mutated in > 10% of tumors in both cohorts. The 3 exceptions (blue) also show high mutational frequency but did not reach the 10% cutoff in one or both other cohorts.

Common genes mutated in >10% of each cohort			
**Gene**	***EGFR*-mut n=58**	***KRAS*-mut n=163**	**WT n=313**
TP53	34	57	177
MUC16	12	79	133
RYR2	10	66	125
FLG	6	39	93
CSMD1	7	46	65
PCLO	6	30	66
AHNAK2	6	17	51
GRIN2A	6	13	32
PKD1L1	6	9	36
LAMB4	6	11	28

Table S3. Conserved genes in the *TP53*-mut interactome across cohorts.

Conservation of *TP53*-mut interactome genes		
***EGFR*-mut**	***KRAS*-mut**	**WT**
TP53BP1	*TP53BP1*	*TP53BP1*
TP53BP2	*TP53BP2*	*TP53BP2*
RASSF5 (NORE1A)	*RASSF5 (NORE1A)*	*RASSF5 (NORE1A)*
EPN3	*EPN3*	*EPN3*
ESPL1	*ESPL1*	*ESPL1*
REV3L	*REV3L*	*REV3L*
TP73	*BCL2L12*	*PTK2*
BRD8	*PSRC1*	*SALL2*
PP1R13B	*TADA3*	*HERC1*
UBE4B	*WDR33*	*GNL3*
SALL1	*ARMC10*	*PSRC1(DDA3*
LATS2	*BCL2L12*	
SREBF1,	*MYBBPIA*	
TOPORS	*GNL3*	
PSRC1(DDA3)	*PSRC1(DDA3)*	
WDR33		
MYBBPIA		
RIF1		
PTK2		
SALL2		
HERC1		
UBE4B		

Table S4. Mutational frequency in members of *KRAS*-mut and *EGFR*-mut interactomes.

Conserved members of reported *EGFR*-mut and *KRAS*-mut interactomes			
**Gene**	***EGFR*-mut N=58**	***KRAS*-mut N=166**	**WT N=313**
	EGFR-mut Interactome		
ENGASE	0	3	6
NDUFA4	0	0	1
AP2A2	0	1	6
AP2B1	0	0	2
AP3M1	0	0	2
ERBB2	0	2	9
CCDC37	0	2	3
DNAJA2	0	0	5
GRB2	0	3	4
HSPA5	0	1	2
UBASH3B	1	2	3
SLC26A3	1	3	13
STAT3	1	2	5
TUFM	1	2	3
	KRAS-mut interactome Interactome		
WDR20	0	0	4
VTI1B	0	0	0
SENP1	0	1	0
LRRC59	0	1	2
FAM126A	0	1	4
CANX	2	1	3
PIP5K1A	0	1	2

Table S5. Available demographic data on each cohort.

**Cohort**	**Age (Years)**	**Gender**	**Smoking History**	**Pack Years for Smokers (Years)**	**Stage**
EGFR-mut N=58	65.5 +/- 9.1	43 female<br>12 male	26 lifetime non-smokers<br>27 with smoking history	18.2 +/- 26.6	24 stage I<br>14 stage II<br>12 stage III<br>4 stage IV
KRAS-mut N=167	65.2 +/- 10.4	61 female<br>68 male	8 lifetime non-smokers<br>141 smokers	38.2 +/- 25.1	75 stage I<br>39 stage II<br>27 stage III<br>8 stage IV
NKE N=313	65.5 +/- 10.3	138 female<br>133 male	36 lifetime non-smokers<br>230 smokers	44.8 +/-25.1	164 stage I<br>57 stage II<br>41 stage III<br>13 stage IV
